# The Importance of Data Reliability and Usability When Assessing Impacts of Marine Mineral Oil Spills

**DOI:** 10.3390/toxics9110302

**Published:** 2021-11-12

**Authors:** A. Dallas Wait

**Affiliations:** Gradient, One Beacon Street, 17th Floor, Boston, MA 02108, USA; dwait@gradientcorp.com; Tel.: +1-617-395-5527

**Keywords:** mineral oils, marine oil spills, data quality, data usability, sampling and analysis

## Abstract

Spilled mineral oils in the marine environment pose a number of challenges to sampling and analysis. Mineral oils are complex assemblages of hydrocarbons and additives, the composition of which can vary considerably depending on the source oil and product specifications. Further, the marine microbial and chemical environment can be harsh and variable over short times and distances, producing a rigorous source of hydrocarbon degradation of a mineral oil assemblage. Researchers must ensure that any measurements used to determine the nature and extent of the oil release, the fate and transport of the mineral oil constituents, and any resultant toxicological effects are derived using representative data that adhere to the study’s data quality objectives (DQOs). The purpose of this paper is to provide guidance for crafting obtainable DQOs and provide insights into producing reliable results that properly underpin researchers’ findings when scrutinized by others.

## 1. Introduction

Mineral oils are complex assemblages of hydrocarbons manufactured from crude petroleum [1]. Mineral oil production involves first distilling crude oils at atmospheric pressure and then, under high vacuum, generating distillates and residuals that can be further refined into mineral oils [2,3]. Mineral oils refined from crude oils consist of a mixture of straight and branched-chained paraffinic, naphthenic, and aromatic hydrocarbons within a boiling point range of 300–600 °C [4], with resulting carbon ranges from C15 to C50 [2]. Mineral oil composition and physical characteristics can vary widely depending on the source of the oil and product specifications. Further, base stock mineral oils can be chemically modified into “synthetic” mineral oils [5]. The expansive composition of mineral oils allows for a wide variety of uses [6], which include non-lubricating products (e.g., agricultural spray oils, insulating oils, coatings, and printing inks), lubricating products (e.g., crank case oils and transmission fluids), and highly refined medicinal white oils and “paraffinum perliquidum” [3,7]. Further enhancing the complex composition of mineral oils is the liberal use of additives (“additive packages”), often organo-metallic compounds, including corrosion inhibitors, antioxidants, antifoaming agents, detergents, dispersants, and emulsifiers, which are blended into the mineral oils [8,9]. Plant-based oil spills (e.g., canola, soy bean, corn, palm, and neem oil), which occur more often than one may realize, are not the focus of this paper, but the data quality discussion herein is certainly applicable to investigations of plant-based oil spills in the marine environment.

Petroleum releases into the ocean are significant, resulting from natural seeps as well as spills during oil extraction, processing, transportation, and use [10]. Oil spills can either be accidental or intentional. Accidental spills are most often from tankers transporting crude oil or petroleum products such as mineral oils or, to a lesser extent, are the result of pipeline leaks, coastal facility spills, and offshore oil production facilities [11]. The largest sources of intentional operational discharges include discharges from vessels (e.g., bilge releases, which may include mineral oils) and water discharges from offshore platforms [10]. The presence of petroleum lubricants, i.e., mineral oils, in today’s ships have varied purposes, including engine lubrication, hydraulics control, and the “oiling” of motors and cranes that may find their way accidentally, or intentionally, into the waterways [12]. During the 1990s, the best estimates for the volume of worldwide releases of petroleum annually into the marine environment were 123,000 tons from accidental sources, 352,000 tons from intentional discharge sources, and 600,000 tons from natural seeps [10,11]. The percentage of these releases attributable to mineral oils is unknown. Over the past two decades, oil spill occurrences have lessened due, in part, to improved prevention programs, technological advances such as the use of double-hulled tankers [13,14], and the enactment of legislation such as the Oil Pollution Act of 1990 (33 U.S.C.&2701 et seq. (1990)) [15]. In addition, there have been recent efforts to promote more environmentally friendly mineral oils for marine applications [16]. Nonetheless, significant mineral oil spill risks still exist and cannot be ignored.

The objective of any sampling and analysis program is to determine the representative physical and chemical characteristics of a sample and, in the case of a mineral oil spill, to reliably understand the nature, extent, and impact of the spill [17]. The need for measurements that are reliable, and of known quality is key to any relevant oil spill study and can be an especially challenging undertaking considering the complex and sometimes unknown composition of mineral oils and the rigorous degradative forces encountered in the marine environment [18]. Publications discussing the sampling and analysis of mineral oils in the marine environment are rather limited, hence the impetus for this review and guidance. This paper provides guidance for crafting obtainable data quality objectives (DQOs) as well as insights into producing reliable results that meets the needs of the investigator(s).

## 2. Data Quality Primer

Prior to the 1970s, nearly all advances in analytical chemistry were instigated in academic and research laboratories, with little consideration for mass applications supporting environmental research. The formation of the US EPA in 1970 provided the platform for comprehensive environmental regulations at the federal level. At that point, most environmental test method development activities were driven by regulatory needs, with both technological and quality control (QC) constraints. During the 1970s, test method development and commensurate QC measures were slow to evolve. That changed in the late 1970s with the momentous Love Canal data quality collection activities that highlighted the problems in producing reliable and usable environmental data [19].

Soon thereafter, in 1980, was the emergence of the Comprehensive Environmental Response, Compensation, and Liability Act (CERCLA, aka Superfund) and the Resource Conservation and Recovery Act (RCRA). CERCLA was specifically designed to enforce rigorous document control, chain of custody, quality assurance (QA), and QC procedures for all aspects of sampling and analysis. In turn, the RCRA program began to develop test methods for assessing hazardous waste. The RCRA test methods were assembled into a test method manual known as SW-846. During the 1980s, the SW-846 test methods became more reliable and defensible. For instance, the opening for the third edition of SW-846 in 1986 states:

It is the goal of the U.S. Environmental Protection Agency’s (EPA’s) QA program to ensure that all data be scientifically valid, defensible, and of known precision and accuracy. The data should be of sufficient quality to withstand scientific and legal challenge relative to the use for which the data are obtained [20].

**Figure 1:**
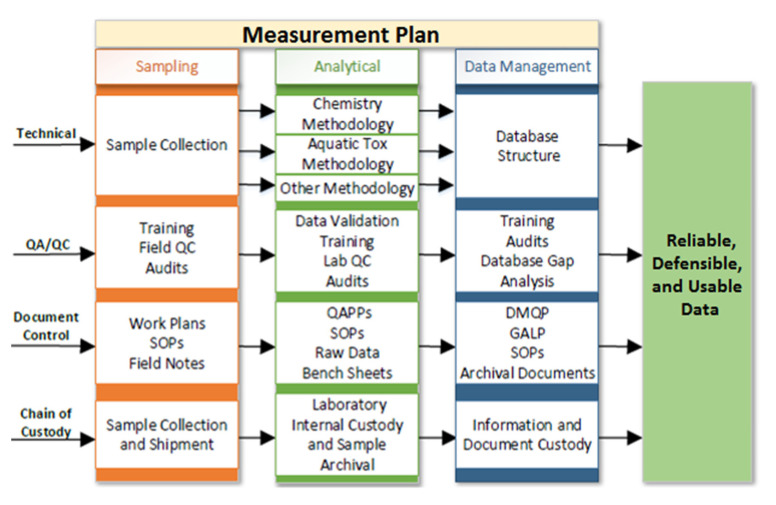
Measurement Plan.

During the 1980s, regulatory method development advanced, but acquiring quality data remained a struggle, even though acceptable and defensible data quality was widely recognized as a cornerstone to the validity of decisions made by environmental managers [21]. The measurement process includes all sampling, analysis, and data management efforts, as shown in Figure 1. There are four simultaneous pathways for conducting a reliable measurement program that include (1) the technical approach, (2) the QA and QC plans, (3) the document control system, and (4) the chain of custody system.

Assessment of errors in a measurement process depends on many levels of details being considered. First, the proper identification of an analyte being quantitated is essential and should not be assumed. Measurable elements include any factors whose impact on the accuracy, precision, and representativeness of a measurement process can be detected, monitored, and quantified by QC samples. Some measurable factors include:

- Blanks, which provide information on possible contamination during sample and analysis activities. Elevated blank levels can lead to higher detection limits and false positives.
- Replicates, which provide information on precision. Data sets with poor replicate precision may not be able to provide confidence in diagnostic forensic evaluations where the data must be evaluated against identification criteria.
- Spikes, which evaluate bias. Samples with out-of-range spike recoveries may be biased low or high relative to the true concentration.

Through the 1990s, regulatory test methods matured and QA/QC practices became well established, culminating in 2000 with the US EPA promulgating an agency-wide Quality System [22]. The elements of the Quality System are the underpinnings for any researcher to ensure their measurements are reliable and usable; this system extends to marine mineral oil spill investigators. Some aspects of a reliable quality system include the development of reasoned DQOs for mineral oil spills, a Quality Assurance Project Plan (QAPP), and supporting standard operating procedures (SOPs) [23,24]. Consideration must also be given to validating measurements and assessing data quality [14].

The measurement process comprises three phases, those being planning, implementation, and assessment [25], and depends on numerous levels of detail being evaluated. Some examples for a marine mineral oil spill are shown in Table 1. The details listed in Table 1 may not be applicable to every marine oil spill but should be considered prior to exclusion.

The underpinnings of reliable and usable data will differ from investigation to investigation depending on the study requirements as defined by its DQOs. Guidance for developing DQOs has been developed by both the US EPA [26] and the American Society for Testing and Materials (ASTM) [27]. DQOs are often misinterpreted with acceptable levels of analytical bias and precision. However, analytical uncertainty is only one aspect of a measurement. DQOs should also consider the uncertainty in health-based standards, forensic tolerances, sample collection, and exposure pathways since each contributes to the overall uncertainty of a decision [28]. Of particular note, many investigators realize that a sample that does not accurately represent study conditions or the population of interest contributes the majority of uncertainty in the data resulting from that sample, which may be as much as 90% [29]. DQOs should include statements about the level of uncertainty that an investigator is willing to accept in the results the study produces.

With this in mind, this paper will now focus on the measurement process being considered for any mineral oil spill investigation, specifically sampling and analysis.

## 3. Marine Mineral Oil Spill Sampling

The objective of a marine mineral oil spill sampling program is to address questions about the marine environment being sampled, for which these questions should be clearly established prior to sample collection. For instance, the researcher may be trying to identify specific contaminants or ratios of contaminants to determine the source, composition, or age of the released mineral oil. Alternatively, a researcher may attempt to define the concentration of the mineral oil or mineral oil constituent within a decision unit to determine the total mass of material discharged or to locate a source by evaluating concentration gradients [19]. To meet these objectives, investigators should consider sampling strategies and sample handling requirements as part of their effort to satisfy the study’s DQOs.

Trying to achieve DQOs for studies conducted in the harsh marine environment (in which mineral oils are subject to, e.g., photolytic reactions, volatilization, biodegradation) can be particularly daunting [30]. Confoundingly, the frequent need for an immediate response to a spill can hamper the planning process. Sampling programs should be crafted to consider, in part, marine background conditions, potential contaminant sources, and inadvertent sample contamination. Background conditions are key to understanding original conditions and can be established using previous long-term monitoring programs, provided they are available in the region and are of adequate data quality. Determining background conditions usually results in additional sampling from unimpacted areas with similar characteristics as the impacted areas or, if the trajectory of an oil spill can be predicted, from pre-impacted areas. In establishing background conditions, anthropogenic and natural sources of the investigation’s contaminants need to be determined. Rigorous methods of sample handling may be needed to eliminate as much cross-contamination and sampling-derived contamination as possible, particularly if the contaminant levels are expected to be low. I have witnessed a number of oil spill investigations where contaminated sampling blanks have precluded the use of the associated results. Contamination from sample collection, processing, preservation, and shipment should be considered, eliminated when possible, and documented when avoidance is not possible.

Contamination introduced during sample collection and processing is cumulative and can be substantially greater than contamination introduced elsewhere in the sample handling and analysis process [18]. Methods for contamination control [31] can include:

- Sample collection techniques, for example:
  ○ proceeding toward a sample location from down current
  ○ progressing from least contaminated areas to most contaminated areas
  ○ circumventing boat exhaust and discharges, which may include mineral oils
  ○ wearing appropriate gloves
- Equipment selection
- Pre-cleaning sampling equipment
- Using contaminant-free and appropriate sample containers
- Decontamination methods for sampling equipment

QC samples are integral to any sample collection investigation and are key in evaluating potential sources of contamination and assessing the reliability of the measured results. The United States Geological Survey (USGS) explains that the goal of collecting QC samples “is to identify, quantify, and document bias and variability in data that result from the collection, processing, shipping and handling of samples” [32]. Further, US EPA states that:

To ensure that the analytical samples are representative of site conditions, QA measures must be associated with each sampling and analysis event. The sampling plan must specify these QA measures, which include, but not limited to, sample collection, laboratory standard operating procedures (SOPs), sample container preparation, equipment decontamination, field blanks, replicate samples, performance evaluation samples, sample preservation and handling, and chain of custody requirements [33].

As such, the study design will define the specific number and types of QC samples needed to meet the DQOs, which should be detailed in the QAPP [34]. QC samples typically collected as part of a well-conceived sampling program include [18]:

- Field, trip, equipment, and decontamination blanks
- Field replicates and duplicates
- Matrix spikes and matrix spike duplicates
- Background samples
- Source materials (mineral oils) potentially spilled, if available

Unlike standard sampling methods that are often available for chemical analysis, standard methods for collecting samples in marine matrices do not exist. When using non-standard sampling methods for marine mineral oil spill research, understanding and applying methodologies used and accepted by other researchers can be key to implementing a reliable sampling program [30]. Accepted practices for sampling mineral oils [35], sediments [36], water column [37], and ecological samples [38,39] have been discussed elsewhere.

## 4. Mineral Oil Analytical Chemistry

The characterization of mineral oils is hindered by its complex assemblage of hydrocarbons and unique additives, of which composition can vary widely depending on the source oil and product specifications. Additives may comprise 10–20% of the mineral oil product [40], which is typically not a consideration when devising a sampling and analysis program for crude oil and refined petroleum spills. As such, additives may prove to be useful as a forensic tool in differentiating mineral oil sources. The testing approach and target analytes will be driven by the DQOs. Broadly, mineral oil spill investigations fall into three categories: (*i*) the initial spill incident investigation, to better understand the direction of the spill and the amount of material spilled; (*ii*) the fate and transport of the mineral oil constituents, with forensic implications as to the who, what, how, when, and where of the mineral oil spill; (*iii*) a toxicological assessment focused on possible human and ecological damages.

Initial marine oil spill incident assessments are often implemented quickly since spills usually occur unannounced and time is of the essence to address the spill once it has occurred. Within the first few days following a spill incident, significant coordination is needed with sampling and analysis activities, which are focused on supporting response decision making. The International Association of Oil and Gas Producers have recently developed guidelines for oil spill incident management and recommendations for emergency response personnel, which may provide valuable insights when developing DQOs [41]. Nonetheless, in accordance with defined DQOs, an appropriate QA program supported by rigorous QC analyses undergirds the production of reliable data and is critical for decision making, even under time-sensitive circumstances [18,42]. Quite often, the types of analyses needed for an initial spill assessment do not require the rigor expected in a sensitive forensic evaluation or toxicological assessment, yet can still be prone to serious errors hampering reliable decision making. For instance, total petroleum hydrocarbon (TPH) measurements can introduce a significantly high bias if plant and algal debris is not adequately considered as a contributor to the TPH results [43]. Particulate coal and wood charcoal can also bias TPH results, particularly in sediment samples.

Fate, source, and transport forensic investigations require rigorous QA/QC programs based on well-crafted DQOs. For example, many forensic studies focus on unique ratios of specific analytes. Known precision and accuracy with tight tolerances are key to producing reliable diagnostic ratios [44]. The minimum laboratory QC measures needed include:

1. Instrument blanks
2. Calibration blanks
3. Method blanks
4. Laboratory control samples (LCSs) (spikes into blank water)
5. Matrix spikes (spikes into site samples)
6. Laboratory replicates

Blanks are indicative of potential contamination issues, replicates provide information about measurement precision, and spikes are a measure of method bias [18]. It is highly recommended, if possible, to analyze performance evaluation samples and reference materials as a measure of method accuracy [18]. A more detailed description of these types of QC measures and their implementation are provided elsewhere [44].

Since crude oils are the fundamental building blocks for mineral oil products, most forensic characterization studies focus on base oil composition using chemical fingerprinting techniques. Further, because of the extensive degradative forces present in the marine environments, such as evaporation, dissolution, “water-washing,” biodegradation, and photo-oxidation, most fingerprinting approaches hone in on resilient polynuclear aromatic hydrocarbons (PAHs) and biomarker compounds [44,45,46,47,48]. The types of PAHs and biomarkers, as well as their ratios and fingerprint patterns, are often considered during the development of DQOs prior to initiating an analytical testing program. The types of biomarker compounds considered in mineral oils typically include tricyclic terpanes (e.g., abietane), steranes (e.g., cholestane), and pentacyclic terpanes (e.g., hopane) [48]. Fingerprinting nearly always involves chromatographic methods, often in combination, such as gas chromatography (GC) with a flame ionization detector (FID), GC with a mass spectrometer (GC/MS), or liquid chromatography with tandem mass spectrometers (LC/MS/MS). Mass spectrometry (MS) methods are often performed in selective ion monitoring (SIM) mode to enhance sensitivity and selectivity. In certain instances, the use of isotopic methods may also be beneficial, such as with a GC isotope ratio mass spectrometer (GC/IRMS) operated in a manner that the isotopic composition of individual compounds in a mixture can be discerned [49]. Recent research using two dimensional gas chromatography (GC × GC) coupled with either an FID or MS has shown promise in further elucidating the composition of complex hydrocarbon mixtures such as that contained in the base oils of mineral oils [50].

As mentioned previously, the collection of background samples and source oil samples are key to any successful marine mineral oil spill investigation, particularly for a forensic sampling and analysis program, and should be integral to the development of DQOs for the investigation. Obtaining and analyzing mineral source oils can be fruitful for providing useful fingerprint information for forensic identification [46]. For example, different mineral oil products can have various additives that are unique to their formulation or have differing concentrations of specific contaminants.

Toxicological assessments take a different tack than those of a forensic investigation but still rely on sensitive, reliable data defined in the DQOs. The four most prominent exposure pathways to be considered are (*i*) dermal exposure to biota, (*ii*) ingestion, (*iii*) partitioning of dissolved hydrocarbons into the tissues of aquatic biota, and (*iv*) inhalation of volatile hydrocarbons [51]. Most petroleum-based oil spills, such as mineral oil spills, focus on the potential toxicological effects of BTEX (benzene, toluene, ethyl benzene, and xylenes), PAHs, and alkylated PAHs. These are analyte-specific measurements that need to be representative and reliable to properly determine whether and how a mineral oil spill has affected the environment. Further, a mineral oil spill assessment must also consider the possible presence of additives, typically organo-metallic compounds, when crafting a sampling and analysis program. Since the use of additives could result in a higher metal content, metal analyses may be warranted (e.g., molybdenum, magnesium, zinc). Using Safety Data Sheet (SDS) information is encouraged by the United Nations International Maritimes Organization’s (IMO) International Maritime Code [52]. When available, and if the alleged identity of the mineral oil spilled is known, a review of the SDS should be useful in the development of the DQOs that target analytes specific to a particular mineral oil product. Due to the complex chemistries of mineral oils, another important issue to consider is whether to conduct bioassay studies to better understand the effects from exposure of the target mineral oil and its degradates on biota [53,54,55]. Saltwater fishes, invertebrates, and plants are typically tested. As a result of the diversity of marine biota, hundreds of species have undergone saltwater toxicity testing, yet few tests are considered standard [54]. These standard tests measure either acute toxicity (lethal or immobilization effects that occur over a short period of time) or chronic toxicity (sublethal effects, such as inhibition of fertilization, growth, and reproduction that occur over a longer period of time). Another consideration in constructing a reliable sampling and analysis plan is the unintended harm that oil spill dispersants may cause to the marine ecosystem [56,57]. Dispersants in a mineral oil spill are applied to reduce the hazards of surface oil in both nearshore and offshore habitats. Dispersants increase the amount of oil in the water column as dissolved oil constituents and small droplets. Fish and other species may potentially be exposed through ingestion and/or dermal absorption. The application of dispersants warrants careful consideration when developing DQOs.

## 5. Conclusions

Understanding the ramifications of a marine mineral oil spill can be complex. Representative data of known quality and integrity is key to making scientifically sound decisions that can be defended when scrutinized by others. Marine mineral oil investigators should proactively design sampling and analysis studies with clearly defined DQOs and ensure that the study is performed according to the plan and that its implementation is properly recorded.

## Figures and Tables

**Table 1:** Considerations in Planning a Mineral Oil Spill Measurement Program.

Objectives	Project Details	Sampling	Analytical	Validation and Assessment
Need for program	History	Representativeness	Subsampling	Data quality objectives
Regulations	Waste generation	Health and safety	Analytes	Documentation of quality
Thresholds or standards	Waste handling	Logistics	Preparatory method	Documentation of activities
Protection of human health	Contaminants	Sampling approach	Analytical method	Completeness/representativeness
Environment protection	Fate and transport	Sampling locations and depths	Aquatic toxicity testing	Bias and precision
Liability	Sources of contamination	Number of samples	Matrix/interferences	Audits
Data quality objectives	Areas to study	QA samples	Detection limits	Performance evaluation samples
Company/agency directives	Exposure pathways	Sample volume	Holding/turnaround times	Chain of custody
Public relations	Use of dispersants	Compositing	Contamination	Usability assessment
End-of-use data		Containers/equipment	QC samples	
		Decontamination	Reagents/supplies	
			Reporting requirements	

## Data Availability

Not Applicable.
